# Non-invasive detection of corneal sub-basal nerve plexus changes in multiple myeloma patients by confocal laser scanning microscopy

**DOI:** 10.1042/BSR20193563

**Published:** 2020-10-21

**Authors:** Anita Koschmieder, Oliver Stachs, Brigitte Kragl, Thomas Stahnke, Katharina A. Sterenczak, Larissa Henze, Anselm G. Jünemann, Christian Junghanss, Rudolf F. Guthoff, Hugo Murua Escobar

**Affiliations:** 1 Department of Ophthalmology, Rostock University Medical Center, Doberaner Straβe 140, Rostock 18057, Germany; 2 Department of Medicine, Clinic III – Hematology, Oncology, Palliative Medicine, Rostock University Medical Center, Ernst-Heydemann-Str. 6, Rostock 18057, Germany

**Keywords:** Confocal Laser Scanning Microscopy, Cornea, Corneal Nerve Morphology, Multiple Myeloma, non invasive, Peripheral Neuropathy

## Abstract

Purpose: Confocal laser scanning microscopy (CLSM) is a non-invasive technique for cellular *in vivo* imaging of the human cornea. CLSM screening was evaluated for early detection of corneal nerve morphology changes and neuropathogenic events in different stage multiple myeloma (MM) patients. As MM patients show disease as well as therapy-related neuropathological symptoms, CLSM potentially provides a tool for non-invasive early detection of neuropathogenic events. CLSM findings were compared with the severity of peripheral neuropathic (PNP) symptoms.

Methods: The study enrolled 25 MM patients in which bilateral ophthalmologic examination was performed including unilateral CLSM. Further peripheral nerve function was clinically evaluated using the conventional neuropathy symptom and neuropathy deficit scores (NDSs).

Results: In 18/25 MM patients, CLSM detected atypical morphological appearance of bulb-like enlarged nerve endings in the corneal sub-basal nerve plexus. These neuromas were only found in patients showing moderate to severe PNP, in patients with mild or lacking PNP neuromas were absent.

Conclusions: CLSM provides a novel non-invasive diagnostic tool for identification of neuromas in cancer patients affected by therapy or disease-related neuropathologies, perspectival allowing early neuronal degenerative process detection and monitoring.

## Introduction

Multiple myeloma (MM) is a hematologic malignancy with an incidence of approximately 5 per 100000 people per year [1]. Early clinical symptoms observed with MM patients are unspecific-like pain or fractures due to osteolytic bone lesions. Later, hypercalcemia, renal failure, anemia and sometimes nerve damage are observed. Among these symptoms, peripheral neuropathy (PNP) can cause considerable loss of quality-of-life.

Accordingly, PNP of MM patients has been in focus over the last 10 years. Several studies have shown that up to 54% of MM patients develop PNP either as effect of MM itself (reported frequencies of 1% up to 20%) or as a result of therapeutic intervention [2,3]. The mainstay of therapeutic interventions nowadays are proteasome inhibitors and immunomodulatory imide drugs (IMiDs), in combination or sequential with chemotherapy and monoclonal antibodies. Bortezomib, the first and until now most widely used proteasome inhibitor, is associated with a high frequency of PNP (38% all grades and 6% grade 3/4 according to CTCAEv3) [3]. PNP is also a pronounced and often treatment-limiting side effect of thalidomide, the first IMiD substance used for MM [4]. PNP is seen less frequently (1–5% grade 3/4) when using lenalidomide or pomalidomide [4,5]. According to former studies, PNP caused by therapeutic intervention is dose-dependent with a plateau of affected patients with the fifth cycle of treatment [6], mean time for development of PNP symptoms was found to be 3 months after beginning of therapy [7]. Nonetheless, prediction for the individual patient is not possible so far. The ethiopathology of PNP in MM patients is still unknown. Several mechanisms are currently discussed such as amyloid deposition or cytokine-mediated damage.

Conventional evaluation of PNP in MM patients is performed in routine diagnostics, ideally starting at baseline before therapy, and periodically thereafter, by analysis of sensory as well as motoric nerve fiber function. Currently the gold standard in diagnosis of neuropathy is electrophysiological measurements recording physical parameters as the number of nerve fibers and their conduction velocity. Alternatively, PNP scoring in MM patients might be performed analogous to PNP scoring in diabetes mellitus (DM), using clinical scores as the neuropathy symptom score (NSS) and the neuropathy deficit score (NDS) [8] focusing on clinical evaluation of subjective symptoms. Accordingly, a correlation of morphological nerve changes in eyes of MM patients with clinically detectable neuropathy scoring is potentially possible. NSS and NDS graduate PNP symptoms into three grades, from mild to moderate to severe. Clinical assessment of neuropathic degeneration with conventional assessment scores is often performed at a pathogenic stage in which the severity of PNP is already affecting the patients’ quality of life. Accordingly, novel methods allowing for early detection of neuropathic degeneration are of considerable interest for MM patients. Future studies using CLSM may allow the evaluation of nerve fiber damage in the SNP under different treatment regimens for evaluation of the least neurotoxic effects with corresponding study designs including control groups.

Confocal laser scanning microscopy (CLSM) is a non-invasive *in vivo* imaging method of the human cornea, delivering detailed images of cellular layers as well as the architecture of the corneal sub-basal nerve plexus. This technique has lately been used in DM type 2 to evaluate neural degeneration in cornea as an early neuropathic marker. These studies showed that changes in morphology and quantity were detectable in the sub-basal nerve plexus with CLSM in DM type 2 patients in early stages even before they were clinically apparent and detectable with conventional methods [9–11]. The confocal microscopy qualifies, by its non-invasive character, as an interesting alternative or additional screening tool for neuropathic processes. Herein, we describe a first CLSM screening of different stage MM patients in order to evaluate if CLSM can be used to characterize and describe changes of the corneal nerve morphology as well as an identification of peripheral neuropathic (PNP) symptoms in MM patients.

## Methods

### Patients

The aim was to analyze if CLSM can be used to identify changes in corneal nerve fiber morphology in a representative group of MM patients. Our study was designed as a cross-sectional study including patients diagnosed with MM in different stages of their disease and treatment. In total, 25 patients diagnosed with MM were included. The recruitment of patients was coordinated with the Study Office at the Department of Hematology, Oncology, Palliative Medicine at Rostock University Medical Center. Exclusion criteria were DM, dry eye syndrome, hypovitaminosis and severe chronic nephropathy. The study was designed as a cross-sectional study to investigate changes in corneal nerve morphology and changes of peripheral neuropathy symptoms at different stages of the disease. Approval of the applicable Ethics Committee of the University of Rostock in accordance with applicable laws, rules and regulations was obtained in January 2015. Recruitment started in December 2015 and is still ongoing. All performed examinations and experiments were also in accordance applicable laws, rules and regulations of the University of Rostock. Every patient who signed informed consent was included. Patients were included regardless to the timepoint of their initial diagnosis and their previously received therapy. CLSM screening and clinical peripheral neuropathy screening was performed in the Department of Ophthalmology at Rostock University Medical Center.

### Ophthalmologic examination

The ophthalmologic examinations of all patients included: measurement of the intraocular pressure, visual acuity, slit lamp examination of the cornea, tensio (non conact), break-up-time (to rule out Sicca syndrome), optical coherence tomography of the cornea followed by unilateral confocal microscopy (CLSM) of the cornea. The method is specified below.

### CLSM screening

The patients’ corneas were investigated *in vivo* using the Heidelberg Retina Tomograph (HRTII) in combination with the Rostock Cornea Module (RCM) (Heidelberg Engineering, Heidelberg/Germany). The HRTII/RCM system uses a diode laser source with a wavelength of 670 nm and is equipped with a water contact objective (63×/0.95 W, 670 nm; Zeiss, Jena/Germany). The distance from the cornea to the microscope was kept stable by a single-use contact element in sterile packaging (Tomo-Cap; Heidelberg Engineering, Heidelberg, Germany). Coupling between the patient’s cornea and the cap was facilitated with a thin lubricant layer of Vidisic gel (Bausch & Lomb/Dr. Mann Pharma, Berlin/Germany; refractive index 1.35). The eye to be examined was anaesthetized by instilling Proparakain 0.5% eye drops (Ursapharm, Saarbrücken/Germany). Image acquisition of the central cornea was performed in z-scan of automatic volume scan mode (30 images, volume depth 60 μm, constant interslice distance 2 μm). The acquired images have a definition of 384 × 384 pixels over an area of 400 × 400 μm. Confocal microscopy was performed in the region of interest, i.e. at the level of epithelium, sub-basal nerve plexus, Bowman’s membrane and anterior stroma at depths from 0 to 150 μm. At least three scans were performed from the central and mid-peripheral corneal region. The total duration of *in-vivo* CLSM was ∼15 min.

### Nerve function testing

Nerve function sensitivity was clinically evaluated using the neuropathy scoring measures NSS and the NDS [12]. The assessment included specific questions regarding neurological symptoms (sensory, motor) with particular attention on neuropathic pain occurrence, and thermoregulatory disturbance (burning sensation or coldness) and measuring sensation in the feet and legs through tuning fork, monofilament, temperature, as well as the Achilles tendon reflex. These scores graduate the degree of peripheral neuropathy into three grades: mild, moderate and severe (Tables 1–3). Corneal sensitivity was tested with Cochet–Bonnet Esthesiometer [13].

**Table 1:** NSS

NSS		
	Yes	No
Symptoms feet, lower leg		
Burning sensation, numbness, paresthesia	2	0
Weakness, spasms, pain	1	0
Localization		
Feet	2	
Lower leg	1	
Elsewhere	0	
Exacerbation		
Only present at night	2	
Present at day and night	1	
Only at day	0	
Patient gets awake by symptoms	1	
Improvement of symptoms while		
Walking	2	
Standing	1	
Resting (sitting or laying)	0	
Total score (0–10)		

**Table 2:** NDS

NDS		
Achilles tendon reflex	Right foot	Left foot
Normal	0	0
Reduced	1	1
Absent	2	2
Perception of vibration (tuning fork on big toe joint)	Right foot	Left foot
Normal	0	0
Reduced or absent	1	1
Perception of pain (monofilament on dorsum of the foot)	Right foot	Left foot
Normal	0	0
Reduced or absent	1	1
Perception of temperature (at dorsum of the foot)	Right foot	Left foot
Normal	0	0
Reduced or absent	1	1
Total score (0–10)		

**Table 3:** Graduation of PNP with NSS and NDS

Graduation of PNP with NSS and NDS		
Total score	NSS	NDS
No PNP	0–2	0–2
Mild PNP	3–4	3–5
Moderate PNP	5–6	6–8
Severe PNP	7–10	9–10

## Results

In total, 25 eyes of 25 MM patients (11 female, 14 male) were examined by CLSM. Patient age ranged from 50 to 78 years. The detailed overview of CLSM scanning time points for each patient’s individual examination is given in months after initial diagnosis in Table 4.

**Table 4:** Appearance of neuroma in CLSM and neuropathy scores evaluated in 25 patients with MM

Patient	Sex	Age	Time in months after diagnosis	Neuroma in CSLM	NSS [0–10]	NDS [0–10]	Additional ocular findings
1	M	50	6	-	0 – no PNP	0 – no PNP	-
2	M	57	1	-	4 – mild PNP	4 – mild PNP	-
3	M	65	3	+	7 – moderate PNP	3 – mild PNP	-
4	F	64	14	-	0 – no PNP	4 – mild PNP	-
5	F	68	6	+	5 – moderate PNP	3 – mild PNP	-
6	M	71	3	+	0 – no PNP	8 – moderate PNP	-
7	M	69	3	-	4 – mild NP	4 – mild PNP	Optic disc edema
8	M	53	92	+	6 – moderate PNP	5 – mild PNP	Optic disc edema
9	F	72	40	+	5 – moderate PNP	5 – mild PNP	-
10	F	71	144	+	8 – severe PNP	6 - moderate PNP	-
11	M	68	6	+	7 – severe PNP	4 – mild PNP	-
12	F	61	6	+	8 – severe PNP	3 – mild PNP	-
13	M	52	3	+	5 – mild PNP	2 – no PNP	-
14	F	67	0	+	0 – no PNP	4 – mild PNP	-
15	F	66	168	+	10 – severe PNP	10 - severe PNP	ocular pain
16	M	77	104	+	8 – severe PNP	8 – severe PNP	-
17	M	63	2	-	0 – no PNP	0 – no PNP	-
18	M	78	1	+	0 – no PNP	2 – no PNP	-
19	F	60	6	+	8 – severe PNP	6 moderate PNP	-
20	M	72	5	+	8 – severe PNP	4 – mild PNP	-
21	F	53	6	+	0 – no PNP	4 – mild PNP	-
22	M	69	48	+	8 – severe PNP	3 – mild PNP	-
23	M	51	1	-	0 – no PNP	2 – no PNP	-
24	F	63	4	+	0 – no PNP	3 – mild PNP	-
25	M	74	0	-	0 – no PNP	3 – mild PNP	-

### NSS and NDS

Values of the two clinically tested neuropathy scores, the NSS and the NDS are shown in Table 3. Score values ranged between 0 and 10, respectively. According to the scoring system only 4 of 25 patients did not show any peripheral neuropathy. All other patients ranged from mild to severe peripheral neuropathy.

### *In vivo* confocal microscopy

**Figure 1:**
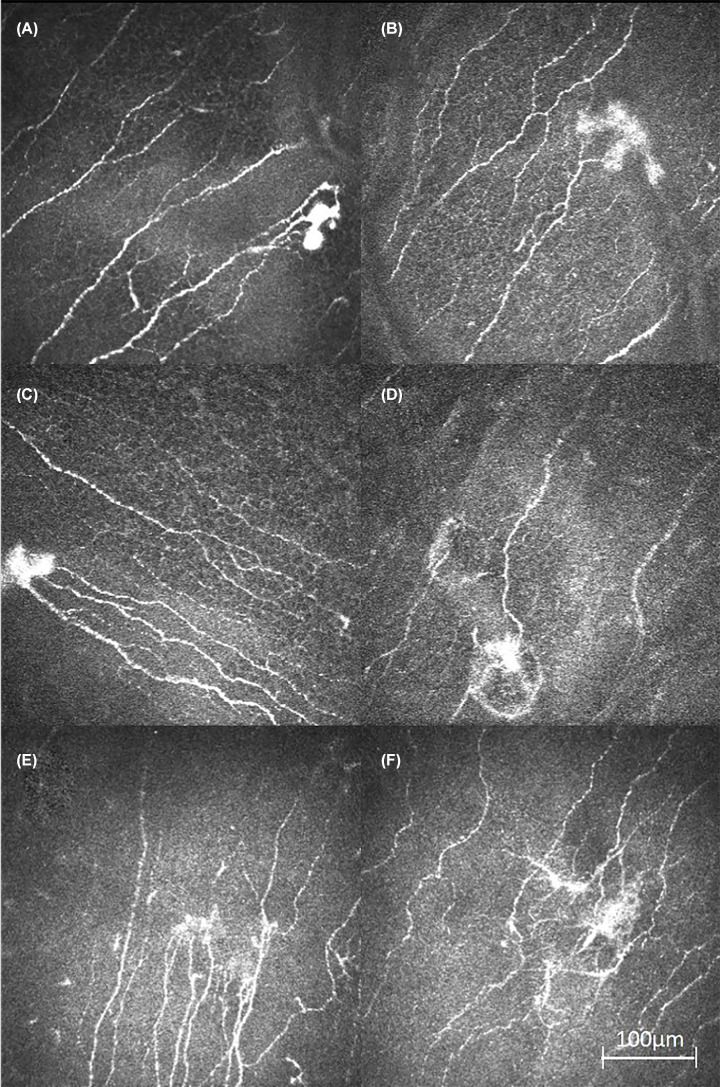
Examples of neuroma formations at the level of corneal sub-basal nerve plexus All pictures are showing a hyperreflective enlargement and bulb-like formation at the ending or a branch of a sub-basal nerve. (**A**) Patient 20; 5 months after initial diagnosis. (**B**) Patient 6; 12 months after initial diagnosis. (**C**) Patient 8; 104 months after initial diagnosis (basal epithelial cells are seen as a mosaic-like pattern around the nerves in this image plane). (**D**) Patient 3; 3 months after initial diagnosis. (**E**) Patient 12; 6 months after initial diagnosis. (**F**) Patient 24; 4 months after initial diagnosis.

In 18 of 25 patients an atypical morphological appearance of bulb-like enlarged nerve endings, herein called neuroma, was detected in the corneal sub-basal nerve plexus (Figure 1). The seven patients in which no neuroma was observed by CLSM, showed either mild or no PNP. NSS and NDS were ranging from 0 to 4. In all of the patients showing moderate to severe neuropathy scores, with either NSS and/or NDS ranging from 5 to 10, neuroma formations in the sub-basal nerve plexus with CLSM were observed.

### Ocular symptoms and findings

None of our patients presented with a corneal pathology, which was visible with slit lamp examination. All patients showed normal corneal sensitivity tested with a Cochet–Bonnet esthesiometer. Twenty-four of twenty-five patients did not experience any ocular neuropathy symptoms. One of the 25 patients who additionally showed epithelial cysts experienced ocular surface pain. Two of 25 patients presented with an asymptomatic swelling of the optic disc.

## Discussion

The most substantial finding by applying CLSM in patients with MM were formations of bulb-like enlarged nerve endings, herein referred as neuromas. The term neuroma refers to descriptions by Belmonte et al. [14] as a dilatation of an injured sub-basal nerve axon after surgical trauma. He described a thickening of the remaining stump of the central nerve and interpreted this phenomenon by dysregulated hyperregeneration and by proliferation of glia and connective tissue cells around the nerve ending. Those abnormally shaped sub-basal corneal nerve formations, called neuromas, are known to be associated with an abnormal electrochemical responsiveness due to an altered expression of ion channel proteins.

The mechanisms leading to neuroma formation without surgical trauma are not yet well understood. Dua et al. are outlining the anatomical feature of thickened terminal nerves appearing as bulb-like structures at their perforation site through Bowman’s layer. Morphology was described using stained histopathological core sections using acetylcholinesterase staining. The terminal nerve bulb itself was further described as a normal finding in healthy corneas [14]. So far, there is no distinct description of similar findings with *in vivo* confocal microscopy in the healthy human cornea. Abnormal enlargement of these bulb structures with a diameter of up to 24 µm were described in corneal buttons of patients with advanced keratoconus and in bullous keratopathy [15,16]. Apparently, the higher resolution of histological staining allows to identify normal structures that *in vivo* are only visible when pathologically enlarged. Therefore, the visibility of neuromas in CLSM images can be interpreted as a pathologic finding.

Besides in advanced keratoconus and bullous keratopathy, neuroma formations have been described with CLSM in severe dry eye corneal neuropathy. Several authors outlined a connection between the presence of corneal neuromas and neuropathic ocular surface pain [17–20].

The perforation site of sub-basal nerves through Bowman’s layer might be considered as a vulnerable region to develop neuroma formations under pathologic conditions. A reason for morphological transformation, allowing to detect the usually only histologically identifiable bulb-shaped elements by *in vivo* CLSM could be axoplasmatic flow alterations. An *ex vivo* CLSM investigation of corneal nerves post mortem supports this assumption. Dua et al. were able to observe similar bulb-like terminal nerve fiber endings in corneal tissue which developed during the first 5 days after death and explained them as a failure of active axoplasm transport (dying-back) [21].

Therapeutic agents used in the treatment of MM are known to induce neurotoxic side effects, although the exact pathomechanisms are not yet identified. Evidence observed in a rat model suggests that bortezomib, a broadly used substance with MM patients, causes alterations of the nerves cytoskeleton leading to axoplasmatic transport problems [22]. None of the herein examinated patients suffered a loss of corneal nerve sensational function and only one experienced ocular surface pain. In the latter particular case, an additional epithelial pathology was observed with confocal microscopy, but not clinically in slit lamp microscopy. The finding differs in appearance from the well-known entity of bullous keratopathy, in which an epithelial edema can be clinically detected. Such a bullous keratopathy was absent from our patient. In our only case of ocular surface pain, the epithelial cyst formation is the likeliest reason for ocular surface pain.

Despite the absence of local ocular neuropathic symptoms (sensitivity loss and ocular surface pain) most of the patients (18 of 25) developed a clinically detectable peripheral neuropathy (as shown in Table 4).

To interpret the finding of neuroma, we distinguish between the morphological alterations within the corneal sub-basal nerve plexus and the occurrence of neuropathic symptoms. Herein we differentiate between ocular and PNP symptoms. Unlike the findings of Hamrah et al., Patel and McGhee, and Jacobs et al. [17–20], we found no connection between neuroma and neuropathic ocular surface pain in our patient group. However, we noted concurrent occurrence of corneal neuromas and peripheral neuropathy.

Aragona et al. previously used *in vivo* confocal microscopy in patients with MM [23]. Their study group contained 16 newly diagnosed and untreated patients with MM. They detected a significantly higher nerve fiber density with MM patients compared with the healthy control group. They also detected a significantly higher reflectivity of cellular cytoplasm, of basal epithelial cells and anterior stroma keratocytes. Neuroma formations of sub-basal nerves have not been described in their work. The absence of neuromas in their results might be due to another selection of image stacks or to differences in the employed CLSM instrument (Nidek vs Heidelberg), which may lead to different resolutions, making neuroma detection more difficult. Most notably, Aragona et al. examined exclusively untreated patients, while the herein presented study included patients in different stages of disease with the majority undergoing treatment [23]. Further, neuropathic symptoms were not focused within the analyzed patient cohort of Aragona et al. [23].

Several other systemic diseases are known to cause symptoms of peripheral neuropathy. In two of them, DM and terminal nephropathy, confocal microscopy demonstrated morphological changes in the corneal sub-basal nerve plexus that are related to PNP symptoms. In both groups, the specific pattern that correlated to PNP was a reduction of nerve fiber density in the corneal sub-basal nerve plexus. There was no evidence of enlarged nerve fiber endings (neuromas) in patients with DM or terminal nephropathy [9–11].

We consider, that different pathogenesis of peripheral neuropathy in different systemic conditions might lead to different morphological patterns that can be observed with *in vivo* CLSM.

Due to significant dysmorphology of nerve fibers, additional algorithms in automated nerve fiber detection analysis are necessary to minimize artifacts interfering with the automated detection in CLSM images [24]. Therefore, quantitative measures of the sub-basal nerves, such as nerve fiber density and length as well as number of nerve fiber branches, were not analyzed in our study group. By subjective evaluation the nerve fiber density and branching pattern appears to be normal. We are aware that this assumption must be qualitatively proven.

However, in MM patients, the combined clinical assessment of PNP in parallel with the investigation of morphological nerve fiber changes using *in vivo* CLSM is a new approach. We can state a parallel incidence of clinical PNP symptoms and the presence of neuroma formations detected in the corneal sub-basal nerve plexus (18 of 25 patients), without ocular neuropathic symptoms. At this stage we cannot state whether the morphological nerve fiber changes are causally correlated to the condition of monoclonal gammopathy itself, or to the treatment the patients received. To gain a deeper knowledge of their relationship, a larger patient cohort, defined therapeutic treatments and CSLM screening time points in a prospective study design would be recommended. At present, a link between the appearance of corneal neuroma and MM stage or a specific therapy regime cannot be conclusively provided. Moreover, given the recently available image analysis tools, it is not possible to quantify neuroma patterns. This is due to its high variability as well as to the complexity of the structures. Therefore, at present we have to limit ourselves to a qualitative description. However, herein we report for the first time application of CSML in a MM patient cohort undergoing myeloma treatment. The identification of the described neuromas shows that CSML serves as a highly sensitive tool to characterize morphological neuronal changes in MM patients.

## Conclusions

CSLM provides a novel non-invasive diagnostic tool for identification of neuromas in cancer patients affected by therapy or disease related neuropathologies, perspectival allowing early neuronal degenerative process detection and monitoring. In future studies, device developments introduced by Bohn et al. [25] will allow the assignment of pathological structures such as dysmorphologies and neuromas to a distinct depth within the whole cornea.

## Data Availability

The datasets generated during and/or analyzed during the current study are available from the corresponding author on reasonable request.

## Abbreviations

CLSM: confocal laser scanning microscopy
DM: diabetes mellitus
HRTII: Heidelberg Retina Tomograph
IMiD: immunomodulatory imide drug
MM: multiple myeloma
NDS: neuropathy deficit score
NSS: neuropathy symptom score
PNP: peripheral neuropathy
RCM: Rostock Cornea Module
